# Immunomodulatory Activity of *Lactococcus lactis* A17 from Taiwan Fermented Cabbage in OVA-Sensitized BALB/c Mice

**DOI:** 10.1155/2013/287803

**Published:** 2013-01-21

**Authors:** Hui-Ching Mei, Yen-Wenn Liu, Yi-Chin Chiang, Shiou-Huei Chao, Nai-Wen Mei, Yu-Wen Liu, Ying-Chieh Tsai

**Affiliations:** ^1^Department of Science Education, National Taipei University of Education, 134, Section 2, Heping E. Road, Da-an District, Taipei 10671, Taiwan; ^2^Institute of Biochemistry and Molecular Biology, National Yang-Ming University, 155, Section 2, Linong Street, Taipei 11221, Taiwan; ^3^Asian Probiotics and Prebiotics Corporation, Room 9B, No. 6-8 Lane 511, Wuzhong Road, Minhang District, Shanghai, China; ^4^GINCARE Int'l Enterprises Co., Ltd., No. 24, Section 3, Chungyang Road, Tu-Cheng District, New Taipei City 23673, Taiwan

## Abstract

From fermented Taiwan foods, we have isolated numerous lactic acid bacteria (LAB) of plant origin and investigated their biological activities. This study aimed to investigate the immunomodulatory effect and mechanism of *Lactococcus lactis* A17 (A17), isolated from Taiwan fermented cabbage, on ovalbumin (OVA)-sensitized mice. Human peripheral blood mononuclear cells were used to verify immune responses of A17 by IFN-**γ** production. Live (A17-A) and heat-killed A17 (A17-H) were orally administered to OVA-sensitized BALB/c mice to investigate their effects on immunoglobulin (Ig) and cytokine production. The mRNA expression of Toll-like receptors (TLR) and nucleotide binding oligomerization domain (NOD)-like protein receptors in spleen cells was analyzed by real-time RT-PCR. Both live and heat-killed A17 modulate OVA-induced allergic effects. B-cell response was modulated by diminishing IgE production and raising OVA-specific IgG2a production, while T-cell response was modulated by increasing IFN-**γ** production and decreasing IL-4 production. The mRNA expression of NOD-1, NOD-2, and TLR-4 was down-regulated by A17 as well. This is the first report to describe a naïve *Lactococcus lactis* A17 strain as a promising candidate for prophylactic and therapeutic treatments of allergic diseases via oral administration. Our results suggest the ameliorative effects of A17 may be caused by modulating NOD-1 NOD-2, and TLR-4 expression.

## 1. Introduction

Lactic acid bacteria (LAB) are generally believed to promote human health. Various beneficial effects of LAB have been reported in the treatment of inflammatory disorders like ulcerative colitis [1], maintenance of intestinal homeostasis [2], and amelioration of atopic dermatitis in infants [3]. Nevertheless, the effectiveness of LAB is variable due to the use of different strains [4, 5].

Allergic disorders, such as allergic rhinitis [6], atopic dermatitis [7], allergic asthma [8], and food allergies [9], have become increasingly prevalent in many countries. These disorders not only affect the individual's life quality but also become a medical burden on society. Allergies are related to the T-helper cell type 2 (Th2) responses both in T cells and B cells. Th2 responses are characterized by the production of certain cytokines including interleukin (IL)-4, -5, -13, and the production of total immunoglobulin (Ig)E, antigen-specific IgE, and IgG1 [10]. Cytokine production is regarded as T-cell response, and immunoglobulin production is regarded as B cell response. Th1 cells can suppress Th2 responses by secreting interferon (IFN)-*γ*, IgG2a, IL-2, and IL-3 [11]. Therefore, to regulate the immune responses by suppressing the Th2- and enhancing the Th1-responses is expected to be helpful in the treatment of allergy and other Th2 dominant disorders and maintaining healthy immune condition.

Numerous studies have proposed that LAB, live or heat-killed, alleviate allergic symptoms by modulating the Th1/Th2 balance toward a Th1 dominant state. Perinatal administration of live *Lactobacillus rhamnosus* GG (LGG) reduced the development of eczema in children with a family history of this atopic disease [12, 13]. Live *Lactobacillus paracasei* KW3110 administered orally to allergic mice revealed antiallergic effects on both Th1 and Th2 cytokines, including IL-12 induction and IL-4 repression [4]. Heat-killed *Lactobacillus casei *strain Shirota (LcS) stimulated IL-12 secretion, which shifted the cytokine production pattern from a Th2 to Th1 predominance and thereby suppressed IgE production [14], IgG1 responses, and systemic anaphylaxis [15]. *Lactobacilli* species are not the only ones that have been shown to be effective. Oral administration of live* Bifidobacterium breve* M-16V suppressed the Th2 immune responses by reducing the serum level of ovalbumin-(OVA-)specific IgE, IgG1, and IL-4 in OVA-sensitized mice [16]. Thus, either live or heat-killed LAB exhibited the capacity to ameliorate both allergic responses in murine models and in humans. However, the mechanism for either live or heat-killed LAB to modulate whether T-cell or B-cell responses remains to be confirmed.

Toll-like receptors (TLRs) and nucleotide-binding oligomerization domain protein (NOD)-like receptors (NLRs) are receptors that detect unique bacterial component and subsequently activate immune responses of host. Peptidoglycan (PGN) and lipopolysaccharides (LPS), the major cell wall components of gram-positive and gram-negative bacteria, respectively, are ligands for TLR-2 and TLR-4. Nucleotide oligomerization domain 1 (NOD-1), one of NLRs, recognizes PGN-related molecules containing the amino acid meso-diaminopimelic acid (meso-DAP) that are produced by most gram-negative and certain gram-positive bacteria [17], while muramyl-dipeptide (MDP) is the ligand of NOD-2. Oral administration of LAB might trigger the immune responses via these receptors. However, little is known concerning the mechanism of LAB on the expression of TLR-2, TLR-4, NOD-1, and NOD-2.

The Chinese have developed various fermented products in distinct areas [18]. The manufacturing processes and ingredients give these fermented foods unique flavors. People utilize local ingredients including tofu, mustard, cabbage, and bamboo shoot to produce diverse fermented food products. However, the community of LAB and other microbes in these fermented foods remains poorly understood, even for popular fermented foods such as kimchi. We have isolated hundreds of LAB from traditional fermented foods with different sources in various regions of Taiwan. Some novel species have been established as well [19, 20]. In this study, we investigated 96 isolated LAB strains to determine the immunomodulatory activity of LAB. Heat-killed LAB was investigated *in vitro* with human peripheral blood mononuclear cells (hPBMCs), and the resultant cytokine production level was evaluated. Among the tested LAB, *Lactococcus lactis* A17 (A17), a strain isolated from fermented cabbage showed profound immunomodulatory potency. In OVA-sensitized BALB/c mice, orally administered live or heat-killed A17, the effects of A17 on Th1/Th2 responses were further investigated.

## 2. Materials and Methods

### 2.1. Chemicals and Reagents

de Man, Rogosa, and Sharpe (MRS) broth was purchased from Difco (Sparks, MD). 3-(4,5-dimethylthiazol-2-yl)-2,5-diphenyl tetrazolium bromide (MTT) and ovalbumin (OVA) were purchased from Sigma-Aldrich (St. Louise, MO). RPMI-1640 culture medium, fetal bovine serum (FBS), L-glutamate, antibiotics (penicillin, streptomycin, and amphotericin) were obtained from (Gibco BRL, NY). All other chemicals were purchased from Merck (Darmstadt, Germany).

### 2.2. Isolation and Preparation of A17

LAB strains were isolated from Taiwan fermented vegetables [19]. *Lactococcus lactis* A17 was isolated from Taiwan fermented cabbage. Gram's stain, catalase assay, Random Amplification Polymorphic DNA-Polymerase Chain Reaction (RAPD-PCR) banding pattern comparison, and 16S rDNA sequence analysis are carried to identify A17 [20]. *Lactobacillus rhamnosus* GG (LGG) and *Lactobacillus casei *strain Shirota (LcS), used as positive controls, were originally obtained from commercial products and were confirmed by 16S rDNA sequence analysis. LAB was inoculated in MRS broth, cultured at 30°C for 48 h. For live LAB preparation, pelleted bacteria were washed twice with sterile phosphate buffered saline (PBS) and then resuspended to a final concentration of 10^10^ CFU/mL in PBS. As for heat-killed LAB preparation, 10^10^ CFU/mL of LAB were heat-killed at 100°C for 20 min as experimentally required and were stored at −20°C until use.

### 2.3. Human Peripheral Blood Mononuclear Cell Preparation

Based on a previous report [21] with slight modifications, hPBMCs were isolated from healthy volunteers with no history of atopic disease. In brief, hPBMCs were isolated by centrifugation at 1,500 rpm for 20 min using Ficoll (GE, Uppsala, Sweden). After washing, the hPBMCs were harvested and resuspended in RPMI 1640 culture medium supplemented with 10% FBS, 1% L-glutamate, 100 IU/mL penicillin, 0.1 mg/mL streptomycin, and 0.25 *μ*g/mL amphotericin.

### 2.4. Stimulation of Human Peripheral Blood Mononuclear Cells

The effect of LAB on hPBMC cytokine production was performed to evaluate the *in vitro* immunomodulatory activity of LAB. Cell cultures were set up in triplicate in 96-well flat bottom polystyrene microtitre plates. All cultures contained 1 × 10^5^ cells of hPBMCs and 5 × 10^7^ CFU of heat-killed LAB. Heat-killed LGG and LcS [22] were used as positive controls. The plates were incubated at 37°C in 5% CO_2_. The supernatants from the cultures were collected at 48 h and stored at −20°C until used for cytokine analysis. Cell viability was measured using an MTT assay. LAB strains that had a corresponding hPBMC viability exceeding 90% were selected for further cytokine measurements.

### 2.5. Experimental Animals and Feeds

Four-week-old female BALB/c mice were purchased from the National Laboratory Animal Center, Taiwan and maintained in National Yang-Ming University. The animal room was kept on a 12 h light and dark cycle at 25 ± 2°C and 55 ± 15% humidity. The mice were fed a standard laboratory diet (LabDiet Autoclavable Rodent Diet 5010, PMI Nutrition International, Brentwood, USA) to acclimate them for two weeks prior to bacterial feeding. All animal experimental procedures were reviewed and approved by the Animal Management Committee, National Yang-Ming University.

**Figure 1:**
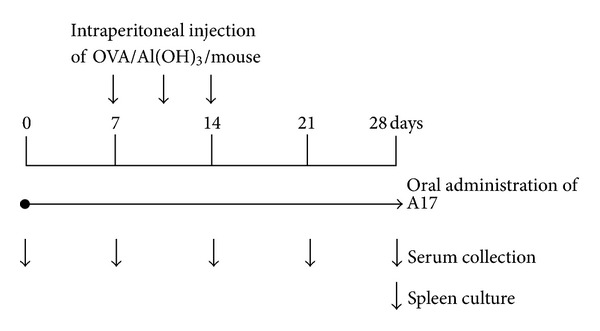
Experimental timeline of the ovalbumin (OVA)-sensitized BALB/c mouse model. Six-week-old female BALB/c mice were fed with live or heat-killed *Lactococcus lactis* A17 (A17) for 4 weeks and intraperitoneally injected three times at days 7, 11, and 14 with 50 *μ*g of OVA in 100 *μ*L of Al(OH)_3_. Serum was collected weekly for immunoglobulin measurement. On day 28, mice were sacrificed and spleens were removed for spleen cell preparation.

To evaluate the effect of A17 on immune responses, the 6-week-old mice were sensitized and challenged with OVA to establish an OVA-sensitized BALB/c mice model as a previous description [15] with slight modifications. The experimental procedure for immunization, administration of LAB, and sample collection in the OVA-sensitized BALB/c mice model is summarized in Figure 1. Four groups (*n* = 8 in each group) of mice were assigned a different bacteria supplement for four consecutive weeks. The healthy control (CON group) and allergy control (OVA group) groups were orally administered PBS by stainless feeding tube. The other experimental groups were orally administered with either live A17 (10^9^ CFU/mouse/day) or heat-killed A17 (10^9^ CFU/mouse/day) by stainless feeding tube. All groups except for the healthy control group were intraperitoneally injected with 100 *μ*L of Al(OH)_3_ containing 50 *μ*g of OVA three times on days 7, 11, and 14. The healthy control mice received Al(OH)_3_ only. Mouse body weight was measured every day during the study period. There were no significant differences in food intake, feed efficiency, or changes in body weight among the groups. Blood was collected using retro-orbital venous plexus puncture and serum was prepared by centrifugation (2,000 rpm for 10 min) weekly starting on day 1 of the experiment. The serum was stored at −20°C before immunoglobulin analysis.

### 2.6. Preparation of Spleen Cells

Mice were sacrificed on day 28 and the spleen cells were harvested for culture according to previous report with modifications [23]. The spleen was ground with sterile flat bottom of a syringe piston to homogenize the spleen cells. The cells were adjusted to 1 × 10^6^ cells/mL in RPMI 1640 medium. In 24-well plates, cells were plated with or without mitogens, such as lipopolysaccharide (LPS, 600 ng/mL) or OVA (25 *μ*g/mL). The plates were incubated in a humidified incubator at 37°C with 5% CO_2_ for 48 h. After incubation, the supernatants were collected and stored at −20°C for further cytokine analysis.

### 2.7. Measurement of Immunoglobulins and Cytokines by an Enzyme-Linked Immunosorbent Assay (ELISA)

The levels of total IgE and OVA-specific IgE, IgG1, and IgG2a were measured using the commercial ELISA kits (Bethyl Laboratory, Inc., Montgomery, AL, USA) [24]. The concentrations of IFN-*γ*, IL-2, IL-4, and IL-10 were determined using ELISA procedure according to the manufacturers' instructions (for mouse cytokines determination, eBioscience, Boston, MA; for human cytokines measurement, R&D Systems, Minneapolis, MN) [25].

### 2.8. Quantitative Real-Time RT-PCR

Total RNA from mice spleen cells were prepared using the TRIzol method (Invitrogen, Carlsbad, CA), and cDNA was then synthesized using the High Capacity cDNA Reverse Transcription Kit (ABI, Foster City, CA). Quantitative real-time PCR was performed in an ABI 7700 Real-time PCR instrument according to the manufacturer's recommendations. Primer sets are listed in Table 1. The housekeeping gene glyceraldehyde-3 phosphate dehydrogenase (GAPDH) was used as an internal control. The expression levels of target mRNAs of each sample were normalized to GAPDH as an internal control.

### 2.9. Statistical Analysis

Data were expressed as means ± the standard deviation (SD). The differences between means were tested for statistical significance using a one-way ANOVA followed by a Tukey's post-hoc test. Differences between the control group and other groups were considered statistically significant when the *P* < 0.05 (*) or <0.01 (**).

## 3. Results

### 3.1. Evaluation of *In Vitro* Immunological Effects of LAB Strains on hPBMCs

**Figure 2:**
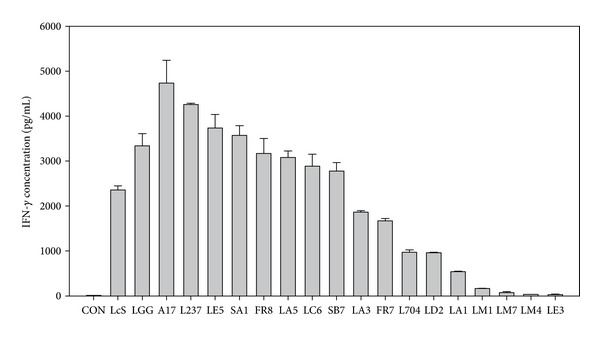
IFN-*γ* production by human peripheral blood mononuclear cells (hPBMCs) stimulated with heat-killed *Lactococcus lactis* A17 (A17), *Lactobacillus casei* strain Shirota (LcS), and *Lactobacillus rhamnosus* GG (LGG). hPBMCs (1 × 10^5^ cells) were cultured with 5 × 10^7^ CFU of heat-killed LAB for 48 hours. Supernatants were collected, and the concentration of IFN-*γ* was determined by ELISA. Each value represents the mean ± SD.

The immunological effect of LAB on hPBMCs was evaluated by measuring the levels of the cytokines IFN-*γ*, which is generally considered to be a Th1 cytokine. LGG and LcS which are commercially available probiotics with recognized immunomodulatory function were used as positive controls in this assay. Heat-killed LAB strains, LcS, and LGG were individually cultured with hPBMCs for determination of IFN-*γ* production. Ninety-six LAB strains from five genera (*Lactobacillus*, *Lactococcus*, *Leuconostoc*, *Pediococcus*, and *Weissella*) including 19 different species were examined. Among the 96 strains, 17 strains treated with hPBMCs and caused the cell viability higher than 90% were further used to determine the IFN-*γ* level.  Figure 2 shows the effects of LcS, LGG, and 17 representatives, A17, L237, LE5, SA1, FR8, LA5, LC6, SB7, LA3, FR7, L704, LD2, LA1, LM1, LM7, LM4, LE3, on the production of IFN-*γ*. According to the results, LcS and LGG groups showing relatively high levels of IFN-*γ* indicated a Th1 dominant response. A17 stimulated the highest level of IFN-*γ* among all tested LAB strains (Figure 2) was furthered investigated the *in vivo* immunomodulatory activities.

### 3.2. Effect of Oral Administration of A17 on Immunoglobulin Expression in OVA-Sensitized Mice

**Figure 3:**
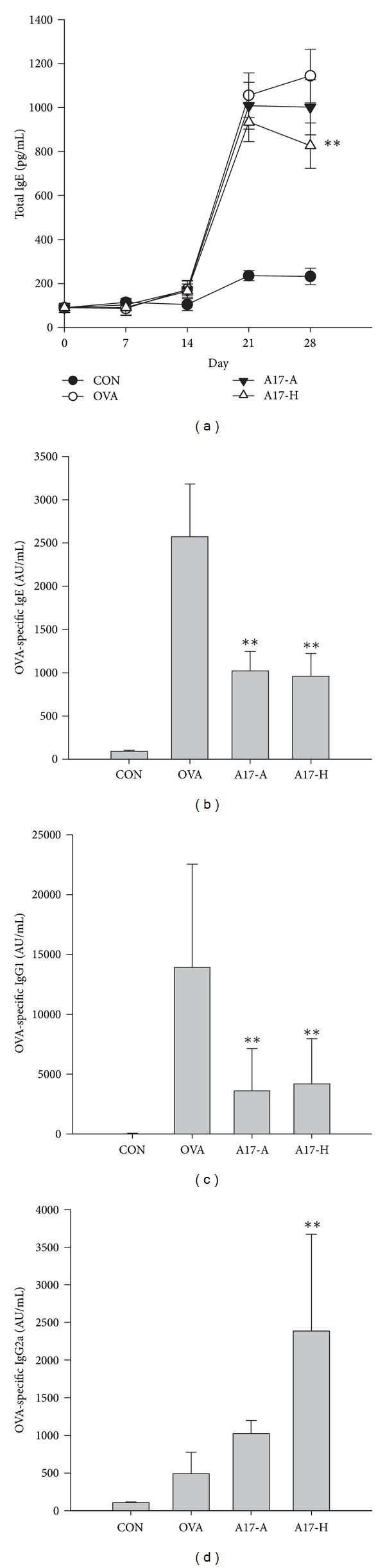
Effect of oral administration of live or heat-killed *Lactococcus lactis* A17 (A17) on immunoglobulins production in OVA-sensitized mouse serum. Six-week-old female BALB/c mice were fed with 10^9^ CFU of live A17 (A17-A) or heat-killed A17 (A17-H) from day 1 to day 28 and intraperitoneally injected three times on days 7, 11, and 14 with 50 *μ*g OVA in 100 *μ*L Al(OH)_3_. Both the healthy control group (CON) and allergy control group (OVA) were administered with PBS orally during the experimental period, and the CON group was not OVA-sensitized. Serum levels of total IgE (a), OVA-specific IgE (b), OVA-specific IgG1 (c), and OVA-specific IgG2a (d) were determined by ELISA. Each value represents the mean ± SD, (*n* = 8). A difference between A17 groups and OVA group was considered statistically significant when *P* < 0.01 (**).

Some LAB strains with Th1 dominant responses were reported to be effective in regulating the production of OVA-induced immunoglobulins [7]. In our study, the suppressive effects of LAB on immunoglobulin E production were analyzed as a preliminary experiment for B-cell response. Mice were orally administered with 10^9^ CFU of either live (A17-A; 10^9^ CFU) or heat-killed A17 (A17-H; 10^9^ CFU) for 4 weeks and intraperitoneally injected with OVA/Al(OH)_3_ on days 7, 11, and 14 (Figure 1). As shown in Figure 3(a), the total serum IgE in OVA-sensitized mice began elevated after day 14 and continuously increased through day 28. Oral administration of heat-killed A17 (A17-H) reduced the serum level of total IgE (Figure 3(a)) and OVA-specific IgE (Figure 3(b)) on day 28 compared to OVA-sensitized group. As for orally administered live A17 (A17-A), the level of OVA-specific IgE (Figure 3(b)) was reduced. A17-H seemed to have a greater IgE suppressive effect than A17-A.

A17-H seemed to have a greater IgE suppressive effect than A17-A. The serum levels of OVA-specific IgG1, Th2-type immunoglobulin, in the A17 groups were significantly lower than in the OVA-sensitized group (OVA) by about 3-fold (Figure 3(c); *P* < 0.01). The reduction in OVA-specific IgG1 amounts among heat-killed and live A17 treatment groups was comparable. A17-H had increased serum level of OVA-specific IgG2a, the Th1-type immunoglobulin, compared with the OVA-sensitized group (OVA) (Figure 3(d)). Interestingly, both heat-killed and live A17 possessed the B-cell responsive ability to reduce Th2-type immunoglobulin production (such as IgE and IgG1) and induce Th1-type immunoglobulin production (such as IgG2a). 

### 3.3. Effect of Oral Administration of A17 on the Cytokine Levels in Spleen Cell Culture from OVA-Sensitized Mice

**Figure 4:**
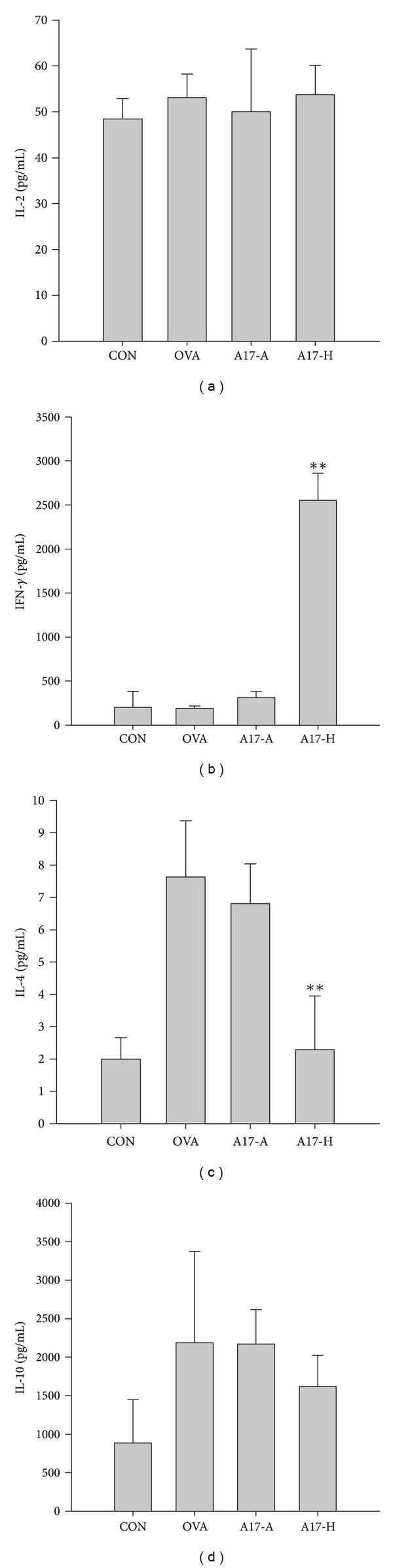
Effect of oral administration of live or heat-killed *Lactococcus lactis* A17 (A17) on cytokines production in OVA-sensitized mouse spleen cell culture. Mice were orally administered with 10^9^ CFU of live A17 (A17-A) or heat-killed A17 (A17-H) from day 1 to day 28 with OVA sensitization during the second week. The healthy control (CON) and allergy control (OVA) mice were administered PBS orally. After sacrificing, mice spleens were removed, and spleen cells (2 × 10^6^ cells/mL) were cultured with OVA (50 *μ*g/mL) for 2 days. The concentration of IL-2 (a), IFN-*γ* (b), IL-4 (c), and IL-10 (d) in the media was determined by ELISA. Each value represents the mean ± SD, *n* = 8. A difference between A17 groups and OVA group was considered statistically significant when *P* < 0.01 (**).

To evaluate the effects of live and heat-killed A17 supplementation on the T-cell responses, the concentrations of IFN-*γ*, IL-2, IL-4, and IL-10 in the supernatant of spleen cell cultures were measured (Figure 4). Spleen cells from mice with OVA sensitization (OVA group and A17 groups) had no significant variation on IL-2 production as compared to healthy control group (CON) (Figure 4(a)). The level of IFN-*γ* in A17-H group was significantly elevated as compared to other groups (CON, OVA and A17-A groups) (Figure 4(b)). As shown in Figure 4(c), the IL-4 level of OVA-sensitized group (OVA) was significantly higher than healthy control group (CON), while in A17 groups, the level of IL-4 in A17-H group was significantly lower than OVA-sensitized group (OVA) and found to be similar to the healthy control group (CON). However, IL-4 production in A17-A was similar to OVA group. The level of IL-10, a regulatory cytokine, was also determined. When IL-10 was measured (Figure 4(d)), the levels of IL-10 were elevated in OVA-sensitized (OVA) and A17 (A17-A and A17-H) groups. These results indicate that heat-killed A17 (A17-H) have a promising effect on modulating the T-cell responses in OVA-sensitized mice.

### 3.4. Effect of A17 on Splenic NOD-1, NOD-2, TLR-2, and TLR-4 mRNA Levels

**Figure 5:**
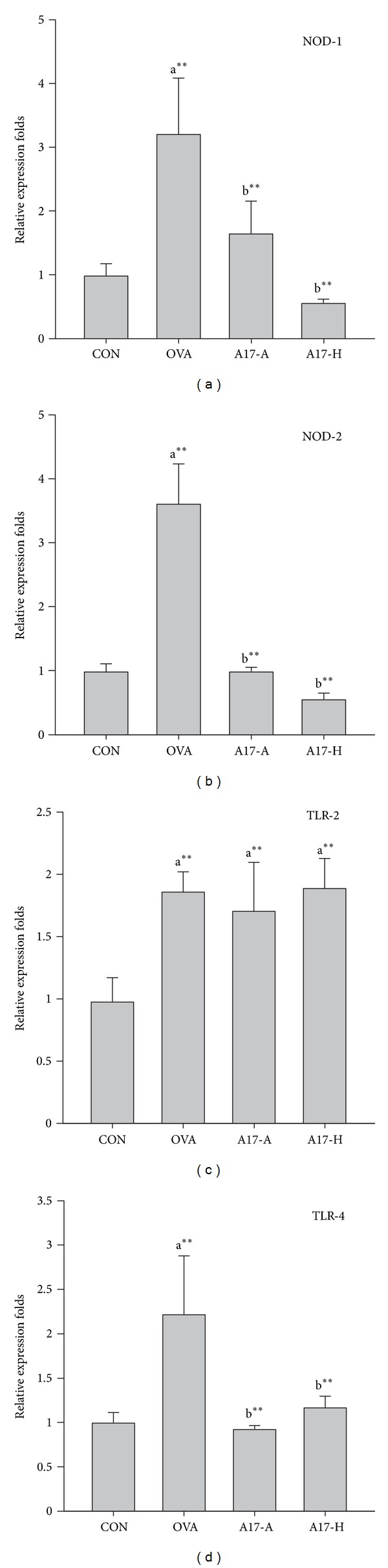
Effect of oral administration of live or heat-killed *Lactococcus lactis* A17 (A17) on mRNA expression in OVA-sensitized mouse spleen cells. Mice were orally administered with 10^9^ CFU of live A17 (A17-A) or heat-killed A17 (A17-H) from day 1 to day 28 with OVA sensitization during the second week. The healthy control (CON) and allergy control (OVA) mice were administered PBS orally. After sacrificing, total RNA was prepared from isolated spleen cells for real-time PCR. The mRNA levels (2^−Δ*Ct*^) of (a) NOD-1, (b) NOD-2, (c) TLR-2, and (d) TLR-4 were determined using quantitative real-time RT-PCR and calculated by subtracting the Ct value of GAPDH from the Ct value of the target gene (Δ*Ct* = *Ct*_target_ − *Ct*_GAPDH_). The values are represented as folds of the healthy control group (CON). The data are expressed as the mean ± SD, with *n* = 8 mice for each group. A difference between comparison groups was considered statistically significant when *P* < 0.01 (**). a, OVA and A17 groups compared with the healthy control group (CON); b, A17 groups compared with OVA group.

To evaluate the expression of TLR and NOD signaling in A17 orally administered mice, the splenic mRNA expression levels of NOD-1, NOD-2, TLR-2, and TLR-4 were examined using real-time RT-PCR (Figure 5). In the OVA group (OVA), the mRNA expression levels of NOD-1, NOD-2, TLR-2, and TLR-4 were elevated as compared to the healthy control group (CON) (*P* < 0.01). When A17 (A17-A or A17-H) was orally administered to OVA-sensitized mice, the expression of NOD-1, NOD-2, and TLR-4 was significantly lower than OVA group (*P* < 0.01). When NOD-1 and NOD-2 were observed, heat-killed A17 (A17-H) showed lower expression level than live A17 (A17-A). However, relative to the healthy control group, both A17-A and A17-H exhibited similar TLR-4 expression. The expression level of TLR-2 was similar in OVA group and A17 groups. These results indicated that OVA sensitization raised NOD-1, NOD-2, TLR-2, and TLR-4 in mice spleen. In live and heat-killed status of A17 OVA-sensitized mice, the mRNA expression of NOD-1, NOD-2, and TLR-4 were diminished.

## 4. Discussion

The relevance of LAB to human health has gained much attention worldwide. Due to the increased incidences of immunological disorders, interest to investigate the anti-influenza virus infection [26], antiallergic [4], and anti-cancer properties [27] of LAB are growing. In the present study, we first reported the relevance of TLRs and NODs on the antiallergic effects of *Lactococcus lactis* A17, a strain isolated from Taiwan fermented cabbage. Most LAB examined in the literature were isolated from animal sources such as milk. In this report, we isolated and investigated the LAB from plant origins. The local components in the decomposed vegetables and fermentation condition make a suitable environment for LAB strains with characteristics various from ordinary fermented materials. We recently reported [25], the anti-inflammatory and immunomodulatory activities of *Lactobacillus plantarum* K68, isolated from a traditional Taiwan fermented food. However, hitherto, few searches concerning the biological effects of LAB from plant-based fermented foods have been reported.

In the present study, immunological effects of LAB strains were evaluated by the cytokine stimulation effects on hPBMCs (Figure 2). Seventeen LAB strains sustain the hPBMC cell viability excess, 90%. This represent that these LAB did not affect the cell viability of hPBMCs; thus, we further determined the IFN-*γ* level. In addition, the levels of tumor necrosis factor (TNF)-*α* were determined to verify the inflammatory status of hPBMCs. Among all tested LAB strains, the levels of TNF-*α*, a marker of the inflammatory response, were comparable to medium as control group (data not shown). Thus, A17 with significant effect on IFN-*γ* production was selected for further investigation. Generally, *Lactococcus lactis* (LL) is accepted safe (GRAS) as a gram-positive bacterium in delivery of therapeutic proteins at the mucosal level [28]. Published research has demonstrated that LL is an efficient host for the production and secretion of heterologous proteins [29]. Some recombinant LL strains have been investigated as agents for treatment of inflammatory disease, allergy, and cancer [30]. Oral administration of recombinant LL expressing bovine *β*-lactoglobulin (BLG), a major cow's milk allergen, partially prevents BALB/c mice from intranasal BLG-sensitization [31]. The development of a mucosal live vaccine using LL as an antigen vehicle has been reported as an attractive and safe vaccination strategy [32]. Recently, LL strains without genetic modification have been shown to possess Th1/Th2 modulatory activity. Intranasal administration of *Lactococcus lactis* G121 isolated from cowsheds of farm was suggested to reduce airway allergic responses and induce a Th-1 polarizing program in dendritic cells in OVA-sensitized BALB/c mice [33]. Neonatal pigs orally treated with *Lactococcus lactis* MG1363 significantly reduced allergic responses to ovomucoid by decreasing the production of IgE and IgG1 [34]. However, the mechanisms for LL strains to modulate immune responses still remain to be clarified.

In the present study, 96 LAB strains isolated from traditional Taiwan fermented food were evaluated for the effect on the *in vitro* production of cytokines by hPBMCs to screen for immunological active strains. *Lactococcus lactis* A17 with elevated IFN-*γ* production, which was grouped as Th1 dominant LAB, was shown to be an antiallergic candidate. Evidences have shown LAB possessed Th1/Th2 regulatory effects. LcS increases the production of Th1 cytokines IL-12 and IFN-*γ* and decreases Th2 cytokines IL-4 and IL-5 [35]. Another antiallergic *Lactobacillus paracasei* strain KW3110 was reported to inhibit the production of Th2 cytokines IL-4, IL-5, and IL-13 [36]. Consequently, we speculated that A17 with an *in vitro* Th1-polarizing potential may exhibit antiallergic effects *in vivo*.

Many studies indicated that the immunogenicity of LAB results from the survival of these bacteria in the gastrointestinal tract since they possess the ability of resistance to gastric acid and adherence to the mucosal surface [37–39]. On the other hand, some studies suggested that nonviable LAB also have immunomodulatory effects [15, 40]. Yoshida et al. [41] reported that live *Lactococcus lactis* strain C59 from dairy starters suppressed IgE production in OVA-sensitized mice via the regulation of IL-4. However, the inhibitory effect on IgE production was lost when heat-killed C59 was accessed in the same model. In our study, the inhibitory effects on the production of IgE were observed in both live (A17-A) and heat-killed (A17-H) A17 groups. Therefore, we speculate that some intracellular or cell wall components of A17 may induce a complex immune-reaction that attribute to the ameliorative effect on OVA-induced responses. We also compared different doses of LAB on modulating the immune responses (data not shown). The results exhibited that both low (10^8^ CFU) and high dose (10^9^ CFU) of heat-killed A17 promoted OVA-specific IgG2a production and suppressed total IgE, OVA-specific IgE, and OVA-specific IgG1 production in dose-dependent manner. Thus, the effects of A17 to regulate the levels of immunoglobulins and cytokines might reach a plateau at relatively low doses of A17. These different observations indicate that diverse routes for immune responses might occur in strain-specific and dose-dependent manners.

TLRs and NODs comprise a family of pattern-recognition receptors that are known to respond to microbial specific patterns [42, 43]. Recently, the expression of NOD-1 and NOD-2 was proved to be necessary for Th2 priming, including T-cell and B-cell responses. NOD-2 was shown to break tolerance to inhaled antigen. This suggested the potential of NOD-2 in driving Th2 lung inflammation [44]. Cytokine expression such as IL-1, IL-4, IL-5, IL-10, and IL-13, in connection with T-cell response, and immunoglobulin G1 (IgG1) production in connection with B-cell response [45], are thought to be related to Th2 immunity as well. As shown in Figure 3, the increasing levels of serum IgE, OVA-specific IgE, and IgG1 in OVA group indicated a B-cell type Th2 responses. Both T-cell responsive Th2 cytokines IL-4 and IL-10 were also increased in OVA group (Figure 4). Moreover, the elevated expression of NOD-1 and NOD-2 in OVA groups represented a raise of Th2 responses (Figure 5). In A17 groups, the levels of IgE, OVA-specific IgE, and OVA-specific IgG1 were significantly lower than in OVA group (*P* < 0.01) (Figure 3). Furthermore, considerable increase of OVA-specific IgG2a was observed in heat-killed A17 (A17-H) group (Figure 3(d)). With regard to cytokine production, A17-H group showed significantly higher IFN-*γ* and lower IL-4 levels relative to OVA group (Figure 4). Meanwhile, the mRNA expression of NOD-1 and NOD-2 in both A17 groups was found to be significantly lower than those in OVA group. Therefore, we suggest that the inhibitory effects of A17 on OVA-induced Th2 responses could be originated from down-regulation of NOD-1 and NOD-2 expression.

Microbial-associated molecular patterns (MAMPs) and danger-associated molecular patterns (DAMPs) appear to be important regulators of adaptive immunity. In terms of MAMPs and DAMPs that activate Th2 immunity; activation of TLR-2 and TLR-4 has been shown to induce Th2 immune responses [46]. Activation of antigen presenting cells by a synthetic ligand of TLR-2, Pam3Cys, resulted in the induction of Th2 associated molecules like IL-13 and M-CSF. Therefore, ligands for TLR-2 were considered to aggravate experimental asthma [47]. TLR-4 signaling is also reported to be required for Th2 priming to antigen [48]. In our results, the expression of TLR-2 in OVA and A17 groups was higher than in health control group (CON), which indicated Th2 immune response was caused by intraperitoneal injection with OVA/Al(OH)_3_. The TLR-4 expression was also elevated in OVA group, while oral administration with both A17-A and A17-H significantly diminished the TLR-4 expression as compared to OVA group (*P* < 0.01). As such, our results further proved the antiallergic effects of A17 owing to repression of NOD-1, NOD-2, and TLR-4 production.

Having established the antiallergic activities of live and heat-killed A17, the existence of some components responsible for the effects are supposed. The active components in other bacteria that induce Th1 cytokines have been reported, such as LPS from gram-negative bacteria [49], lipoarabinomannan from mycobacteria [50], lipoteichoic acid (LTA) from gram-positive bacteria [51], bacterial lipopeptide [52], and unmethylated CpG DNA [53]. These bacterial components are recognized as ligands for different TLRs which trigger cytokine production [54]. In current study, different levels of immunostimulatory activity toward Th1 dominance were exhibited in both live and heat-killed A17. There must be specific features or components that are resistant to heat treatment and are individually recognized by the immune system. As for the diverse immunomodulatory effects, the related researches concerning of live and heat-killed lactic acid bacteria are still limited. Cell wall components such as lipoteichoic acids and peptidoglycans (PGNs) [55, 56] have been considered as factors for the immunomodulatory activities of different bacterial species and strains. A higher correlation has been observed between IL-12p70-stimulatory activity and the amount of peptidoglycan when compared with lipoteichoic acids [56]. However, PGNs seemed not to be responsible for the antiallergic effects of A17 since TLR-2 expression was indistinguishable in OVA and both A17 groups (Figure 5(c)). Ou et al. have compared the cytokine productions of 11 strains of lactic acid bacteria with viable and heat-killed forms in human peripheral blood mononuclear cells. The morphological changes of lactic acid bacteria exposed to heat treatment were observed by field-emission scanning electron microscope (FE-SEM). The cytokine production and changes in cell structure were strain-dependent. It seems that heat treatment may cause changes of the cell components and the morphology of cell surface. In some way the immunomodulatory properties were modified simultaneously [57]. Therefore, the various effects of A17 on NODs and TLRs expression may be caused by different components of A17 which react with immune cells.

In summary, we demonstrated that A17 could ameliorate allergic responses in OVA-sensitized BALB/c mice. A17 induced pronounced immunomodulatory effects on most of the parameters tested. Based on our search, this is the first report on the antiallergic effects of a naïve *Lactococcus lactis* strain A17. Further detailed investigations concern the components, which triggered the immunological mechanism of A17, are in progress. We believe A17 itself or by-products of the cell bodies may be promising candidates for protection from and prophylactic treatment for allergic diseases.

## Figures and Tables

**Table 1:** Primer sets for the real-time RT-PCR.

Gene name	Primer sequence	Size (bp)	Accession number
TLR-2	F: GCTGGAGAACTCTGACCCGCC	217	NM_011905.3
	R: CAAGGATGGCCGCGTCGTTG		
TLR-4	F: AGGAGTGCCCCGCTTTCACC	203	NM_021297.2
	R: TGCCAGAGCGGCTGCCAGA		
NOD-1	F: AGCAGAACACCACACTGACA	141	NM_172729.3
	R: CCTTGGCTGTGATGCGAT		
NOD-2	F: CAGGGACTCAAGAGCAACAC	123	NM_145857.2
	R: GCTGAGCCACTTTAGGTTCT		
GAPDH	F: GTATGACTCCACTCACGGCAAA	101	NM_008084
	R: GGTCTCGCTCCTGGAAGATG		

F: forward primer; R: reverse primer.

## Abbreviations

hPBMCs: Human peripheral blood mononuclear cells
IFN: Interferon
Ig: Immunoglobulin
IL: Interleukin
LAB: Lactic acid bacteria
NOD: Nucleotide-binding oligomerization domain protein
OVA: Ovalbumin
TLR: Toll-like receptor
TNF: Tumor necrosis factor.
